# Model-based quantification of immune response and anti-staphylococcal activity of afabicin in immunocompetent mouse thigh infections to enable predictions of clinical efficacy

**DOI:** 10.1128/aac.00959-25

**Published:** 2026-03-04

**Authors:** Raphaël Saporta, Elisabet I. Nielsen, Annick Menetrey, David R. Cameron, Valérie Nicolas-Métral, Lena E. Friberg

**Affiliations:** 1 Department of Pharmacy, Uppsala University 8097, Uppsala, Sweden; 2 Translational Medicine Department, Debiopharm International SA 463161 , Lausanne, Switzerland; University of California San Francisco, San Francisco, California, USA

**Keywords:** *Staphylococcus aureus*, murine thigh infection model, immune response, afabicin, modeling, pharmacokinetics-pharmacodynamics

## Abstract

This study aimed to describe the immune response and activity of the novel antibiotic afabicin against *Staphylococcus aureus* in an immunocompetent mouse thigh infection model using PKPD modeling and to predict the clinical efficacy of different afabicin dosing regimens in *Staphylococcus*-infected immunocompetent patients. Bacterial counts of five *Staphylococcus aureus* strains were determined over 74 h in an immunocompetent mouse thigh infection model. Afabicin doses of 0.011–150 mg/kg were administered intraperitoneally every 6 h. A PKPD model was developed to describe immune response and afabicin desphosphono (active moiety) activity. The model was used jointly with a human population PK model for afabicin to predict the efficacy of different clinical dosing regimens (intravenous 55 to 160 mg twice-daily or oral 80 to 240 mg twice-daily) in immunocompetent patients. The developed model included a saturable neutrophil-mediated phagocytosis process of bacteria. The afabicin desphosphono effect was characterized using a model structure previously developed based on *in vitro* time-kill and neutropenic mouse thigh infection data. A lower maximum killing rate constant (0.24 h^−1^) and an 82% lower EC_50_ were estimated for immunocompetent animals compared to *in vitro*. Predictions indicated that all tested afabicin regimens would achieve bacterial killing in immunocompetent patients infected with *S. aureus*. Afabicin activity against five *S. aureus* strains was adequately quantified in immunocompetent animals by the developed PKPD model. Predictions supported the efficacy of afabicin 55 mg intravenously twice daily, followed by an 80 mg oral twice-daily regimen in humans, which is currently tested in a clinical trial in bone and joint infections.

## INTRODUCTION

The ability of an antibiotic to be efficacious in treating a bacterial infection depends on using an appropriate dosing regimen. In antibiotic clinical development, dose selection relies on the comprehension of antibiotic pharmacokinetics (PK) and pharmacodynamics (PD) (1, 2), notably through PKPD analyses involving *in vitro* and *in vivo* experimental models (3, 4). The *in vivo* models commonly used to study the PKPD of antibiotics are the neutropenic murine thigh and lung infection models (3–5). Consequently, preclinical data generated during antibiotic development usually do not explore the impact of immune response on bacterial killing or the antibiotic PKPD. Immunocompetent mouse infection models have notably been used in the development of tedizolid, for which dose selection was driven by data from immunocompetent mice based on increased activity in the presence of granulocytes (6, 7). Immunocompetent murine infection models may similarly be valuable for afabicin, an antibiotic that demonstrated activity against intracellular bacteria in phagocytic cells (8).

Neutrophils are the most abundant cells involved in the innate immune response. They contribute to infection control through mechanisms including phagocytosis, in which pathogens are internalized into phagosomes and killed through the activity of antimicrobial agents in the phagosome (9, 10). Previous studies evaluating the impact of neutrophils on bacterial clearance in immunocompetent murine thigh and lung infection models identified a saturable killing process dependent on the neutrophil counts and the bacterial burden (11–13).

Although the translation of preclinical information to recommend clinical doses typically relies on PK/PD indices (14), model-informed drug development (MIDD) approaches have been discussed as an alternative to overcome some limitations of this approach (15–18). PKPD models, usually developed based on *in vitro* and/or neutropenic *in vivo* data, can describe the antibiotic effect on bacteria over time (19–21). However, models describing neutrophil-mediated bacterial killing have rarely been developed (6, 13, 22). Furthermore, these processes have seldom been described simultaneously in a PKPD model (6). Quantifying these processes simultaneously could be valuable to better understand the antibiotic activity in immunocompetent conditions and to inform dose selection in patients.

Afabicin is the prodrug of afabicin desphosphono, which impacts the bacterial fatty acid biosynthesis II pathway (FASII) via inhibition of the enoyl-ACP reductase FabI, an enzyme targeted in staphylococci (23–25), and is in clinical development for staphylococcal acute bacterial skin and skin-structure infections (ABSSSI) and bone and joint infections (BJI) (26, 27). A previous analysis developed a model-based translation from *in vitro* time-kill data to *in vivo* neutropenic mouse thigh infection data for afabicin (28). The current study aimed to integrate data from immunocompetent mouse thigh infection models of afabicin against *Staphylococcus aureus*, originating from the drug development of afabicin, into the model-based translation framework to characterize bacterial dynamics in the presence of an intact immune system and under afabicin treatment, and to use the developed PKPD model to predict the clinical efficacy of different afabicin dosing regimens.

## MATERIALS AND METHODS

### *In vivo* efficacy studies in immunocompetent mice

Immunocompetent female ICR or Swiss Webster mice (*n* = 819) were infected by intramuscular injection of a bacterial inoculum in the thigh. The inoculum size ranged from 6.76 × 10^5^ to 1.26 × 10^8^ colony-forming units (CFU) per thigh in control groups and 6.92 × 10^6^ to 1.15 × 10^7^ CFU/thigh in treatment groups. Inoculum sizes for treatment groups were selected based on the ability to achieve net bacterial growth in control animals. Five *S*. *aureus* strains were evaluated (including one strain evaluated in controls only, Table S1), with an MIC ranging from 0.004 to 0.06 mg/L. MICs were determined using broth microdilution according to CLSI guidelines (M07) (29). Afabicin treatment was initiated 2 h after infection for the non-control groups. Afabicin doses ranging from 0.011 to 150 mg/kg, administered intraperitoneally every 6 h, were evaluated. Mice (up to five planned per group) were euthanized at 2, 4, 6, 10, 12, 20, 26, 50, or 74 h after infection. Thighs were removed aseptically, homogenized, 10-fold serially diluted, and plated on agar. After 20 to 24 h of incubation at 35°C, bacterial counts (in CFU per gram of thigh tissue) were determined.

### Model for immune response

A model was first developed to describe bacterial killing by the immune system in the absence of a drug. The model previously developed for afabicin in neutropenic mouse thigh infections, based on data that included the strains evaluated in immunocompetent mice, was used as a base structure for bacterial dynamics (28). Briefly, the model assumed that bacteria could be in a drug-susceptible and growing (S) state or a dormant (or resting), non-growing, and non-drug-susceptible (D) state (30), with the transfer rate constant from S to D, *k*_SD_, driven by the current bacterial count in relation to the maximum system capacity (*B*_max_). Bacteria were assumed to be in the S state at the start of the experiments. Bacteria-related parameters, i.e., the growth rate constant *k*_growth_, the natural death rate constant *k*_death_, and the maximum system capacity *B*_max_ were fixed, assuming that differences in bacterial dynamics between neutropenic and immunocompetent control groups would be attributable to the immune response only. The equations describing the immune response were developed using all available untreated data. However, parameters related to the immune response were re-estimated jointly with drug effect parameters in further modeling steps, including data from both control and afabicin-treated animals.

Immune-related bacterial killing was initially evaluated as an additional first-order killing rate constant for bacteria in both S and D states. Delays in the immune response were assessed through functions representing a gradual increase in the killing rate constant over time, or through the addition of compartments representing fractions of active and inactive neutrophils.

Alternatively, immune response was characterized through a model accounting for neutrophil dynamics, combined with phagocytosis and digestion processes of bacteria by neutrophils. As neutrophil counts were unavailable, the model structure and parameters for neutrophil dynamics were fixed based on estimates from a previous model-based analysis (22). Neutrophil dynamics (Text S1) were described through a compartment for proliferative cells (*N*_prol_), three transit compartments (*N*_tr1-3_), a compartment for circulating neutrophils (*N*_circ_), and a compartment representing the neutrophil count at the infection site (*N*_thigh_). Recruitment of neutrophils to the infection site was implemented as a surge:


(1) $$\frac{dN_{\text{thigh}}}{dt}=-k_{tr}\cdot \;N_{\text{thigh}}+k_{tr}\cdot \left[1+\frac{N_{\text{amp}}}{{\left(\frac{T-N_{T0}}{N_{sw}}\right)}^{6}+1}\right]\cdot N_{\text{circ}}$$

where *k*_tr_ is the transit rate constant (related to the mean transit time MTT, *k*_tr_ = 4/MTT), *N*_amp_ is the surge amplitude, *N*_T0_ is the peak time, *N*_sw_ is the surge width, and *T* is the time since infection. Although surge-related parameters were initially fixed to literature values (22), a step-wise re-estimation was performed based on control data to explore any infection site-related differences between the studies. A third bacterial state for phagocytosed (P) bacteria was evaluated, with phagocytosis of S and D bacteria determined by the first-order rate constant *k*_phag_. P bacteria were killed by a digestion process described by a first-order rate constant *k*_dig_ (22). Bacterial dynamics in the absence of drug were hence expressed as follows:


(2) $$\frac{dS}{dt}=k_{\text{growth}}\cdot \;S-k_{\text{death}}\cdot \;S-k_{\text{SD}}\cdot \;S-k_{\text{phag}}\cdot \;S$$

(3) $$\begin{array}{c} \frac{dD}{dt}=k_{SD}\cdot S-k_{death}\cdot D-k_{phag}\cdot D \end{array}$$

(4) $$\begin{array}{c} \frac{dP}{dt}=k_{phag}\cdot S+k_{phag}\cdot D-k_{dig}\cdot P \end{array}$$

As CFU counts were obtained after lysing phagocytic cells, non-digested bacteria in the P state were assumed to be quantified in the CFU counts. As killing attributable to phagocytic cells is known to be saturable (11, 12, 22), both the phagocytosis and digestion processes (*k*_phag_ and *k*_dig_) were evaluated to have maximum capacities (*k*_phag,max_, *k*_dig,max_). The current capacities of the processes were tested as dependent on the predicted neutrophil count at the infection site and/or a reduction based on the predicted ratio of phagocytosed bacteria to neutrophils in the thigh (P/N), as exemplified for *k*_phag_:


(5) $$\begin{array}{c} k_{phag}=k_{phag,max}\cdot \left(1-\frac{P/N^{H}}{P/N^{H}+P/N_{50,phag}^{H}\;}\right) \end{array}$$

where P/N_50_ represents the ratio corresponding to a 50% reduction in capacity and *H* represents the sigmoidicity factor. Parameters related to immune response were initially shared between the different bacterial strains, but were also tested to be strain-specific.

### PKPD modeling of afabicin desphosphono activity

The effect of afabicin on bacterial killing in the immunocompetent mouse thigh infection model was analyzed jointly with the previously described *in vitro* data for afabicin desphosphono, and the effect model structure developed for afabicin based on *in vitro* and *in vivo* neutropenic mouse infection data was used (28). The neutropenic mouse thigh infection data previously described were, however, not used in the analysis, as equivalent time points were assessed in immunocompetent mice. Their inclusion would have required additional assumptions regarding neutrophil dynamics under neutropenic conditions.

Unbound afabicin desphosphono (active metabolite) concentrations were derived from a PK model for afabicin in mice (Text S2) (31). The activity of afabicin desphosphono was described by a sigmoid *E*_max_ model, with adaptation compartments representing a reversible reduction of *E*_max_ under antibiotic exposure. The concentration required to reach 50% of *E*_max_ (EC_50_) was scaled using the strains’ MICs, such that:


(6) $$\begin{array}{c} EC_{50}=\beta \cdot MIC^{\gamma } \end{array}$$

where *β* is the scaling slope and *γ* is the scaling power parameter. An afabicin desphosphono effect was only implemented to kill S bacteria, as available data did not support a drug effect on P bacteria.

Drug effect parameters were initially the same for *in vitro* and *in vivo* settings, but later allowed to be separate for the two experimental systems. A lower *in vivo* EC_50_ compared to *in vitro* was previously identified based on neutropenic mouse thigh infection data (28), attributed to a lower β *in vivo*. A parameter β_vivo_ specific to *in vivo* data was thus included, and additional parameters describing differences in effect parameters between *in vitro* and *in vivo* were evaluated by a step-wise inclusion.

### Prediction of clinical efficacy

The developed PKPD model was used for simulations jointly with a population PK model for afabicin in humans, previously built from Phase I data (data not shown, parameters used for simulation in Table S3). Afabicin PK was implemented as a three-compartment model with linear elimination following intravenous (IV) administration, or as a first-order absorption rate with two transit compartments following oral administration. Following conversion from afabicin, afabicin desphosphono PK was described by a two-compartment model, with dose-dependent bioavailability for oral administration (decreasing in bioavailability with increasing doses) and auto-induction of clearance (induction onset half-life ≈19 h).

Afabicin IV administrations of 55, 80, or 160 mg q12h, or oral administrations of 80, 120, or 240 mg q12h, corresponding to doses evaluated in a completed ABSSSI Phase II clinical trial and an ongoing BJI Phase II clinical trial (NCT02426918 and NCT03723551) (27), were simulated in virtual populations of 500 patients with a mean height of 167 cm and a mean weight of 83 kg, corresponding to the covariates included in the PK model, with values sampled from a distribution corresponding to the population ≥18 years of age from the National Health and Nutrition Examination Survey 08/2021–08/2023 data set (32). Inter-individual variability in PK was included in the simulations, whereas inter-occasion variability was not considered. The different doses were evaluated against infections caused by one of the four strains for which afabicin had been assessed in the immunocompetent mouse thigh infection model, with an initial inoculum of 6 to 7 log_10_ CFU/g. Immune response was simulated for patients with a baseline neutrophil count of 1.69 × 10^6^/mL (count equivalent to the mouse baseline neutrophil counts) or 5 × 10^6^/mL (count within the range of normal levels in humans). Parameters related to neutrophil-mediated bacterial killing were assumed to be identical for mice and humans. Treatment was started 24 h after infection, and 72 h of treatment were simulated to coincide with the time of lesion response evaluation, the primary efficacy endpoint in ABSSSI clinical studies (33).

### Data analysis and software

Modeling was conducted in NONMEM version 7.5.0 using the first-order conditional estimation method (34), assisted by Perl-speaks-NONMEM (PsN) version 5.2.6 (35). Bacterial count data in the log_10_ scale were analyzed with an additive residual error using a transform-both-sides approach. Inter-individual variability of PKPD model parameters was not evaluated. Selection between nested models was based on the difference in the objective function value (ΔOFV), where a reduction of 3.84 points (α = 0.05) was required to include an additional parameter. Other model selection criteria included precision of estimated parameters, goodness-of-fit plots (36), and simulation-based visual predictive checks (VPCs) (37). Simulations were performed in R version 4.2.1 (38) using mrgsolve version 1.0.6 (39).

## RESULTS

**Fig 1:**
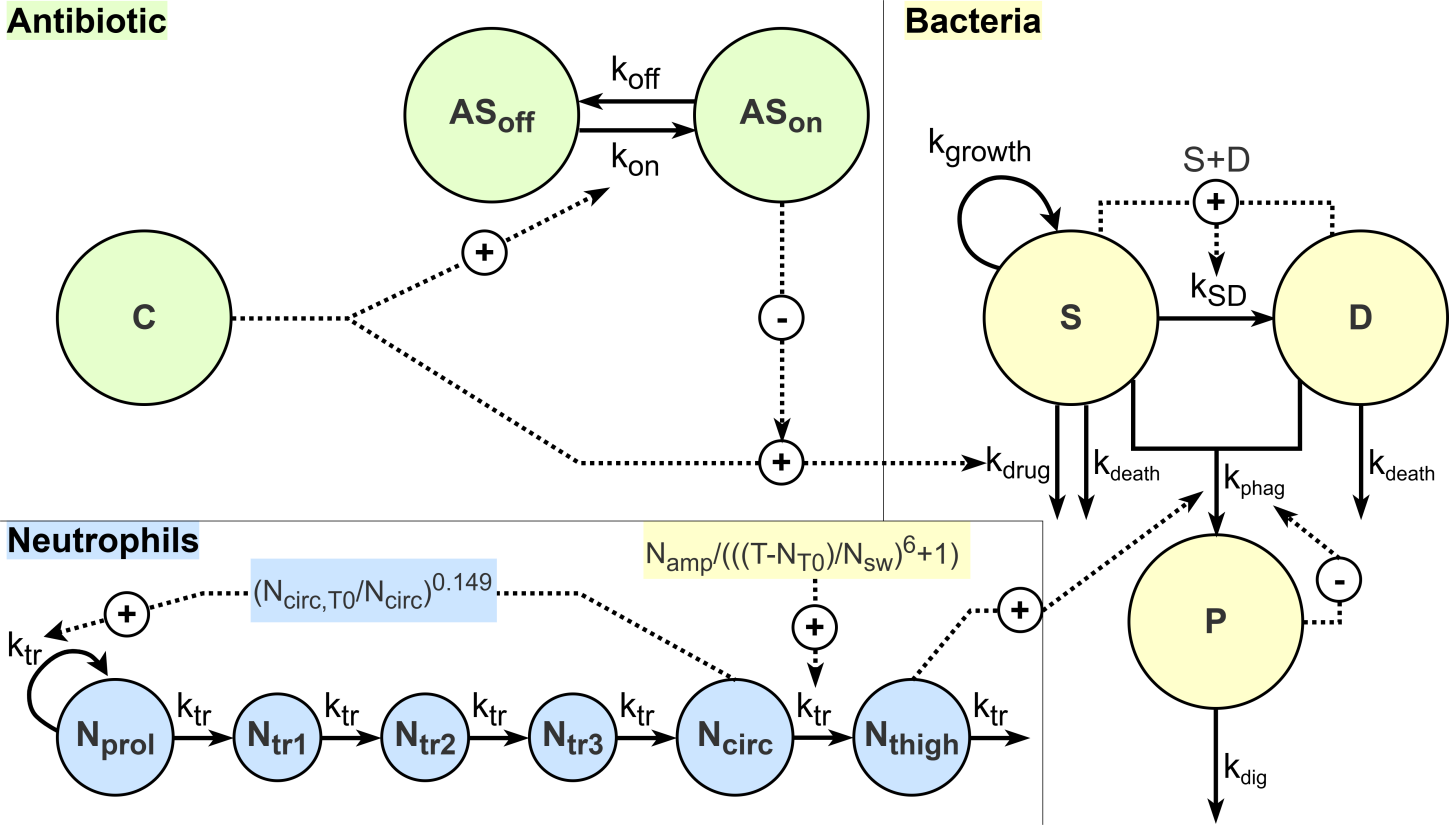
Schematic representation of the final PKPD model. Bacteria are either in a susceptible (S) state, in which they replicate according to the first-order rate constant *k*_growth_, or a dormant (D) state, with bacteria in both states being affected by the first-order natural death rate constant *k*_death_. The transfer from the S to the D state (*k*_SD_) depends on the current bacterial count and the maximum system capacity (*B*_max_). Bacteria in both states can be phagocytosed by neutrophils through a first-order rate constant *k*_phag_, and phagocytosed bacteria (P) are eliminated by neutrophils through a first-order rate constant *k*_dig_. Neutrophil maturation is represented from proliferating (*N*_prol_) to circulating (*N*_circ_) neutrophils through transit compartments (*N*_tr1-3_) and a transit rate *k*_tr_. Neutrophil recruitment to the infection site (*N*_thigh_) is described by a surge of amplitude *N*_amp_, peak time *N*_T0_, and width *N*_SW_. At a given time, *k*_phag_ depends on the maximal phagocytosis rate constant (*k*_phag,max_) and the ratio of P and N_thigh_ (P/N). The afabicin desphosphono unbound concentration (C) induces killing of S bacteria through the first-order rate *k*_drug_. Adaptive susceptibility of bacteria, reducing the maximal drug effect, is represented by a shift from no adaptation (AS_off_) to adaptation (AS_on_) under drug exposure through an onset rate constant *k*_on_, with adaptation reverted through the offset rate constant *k*_off_.

The structure of the developed model for immune response and the afabicin effect is presented in Fig. 1, with parameter estimates and their precision detailed in Table 1. Observed bacterial counts are summarized for representative dose-ranging study groups in Table 2, with results for all studies detailed in Table S2.

**TABLE 1:** Parameter estimates and relative standard errors (RSE) of estimates from the final model

Parameter	Description	Units	Estimate	(RSE %)
Bacterial dynamics				
*k*_growth_	Bacteria growth rate constant	h^−1^	1.10	(Fixed)
*k*_death_	Bacteria natural death rate constant	h^−1^	0.179	(Fixed)
*B*_max vitro_	Maximum system capacity – *in vitro*	log_10_ CFU/mL	9.86	(Fixed)
*B*_max vivo_	Maximum system capacity – *in vivo*	log_10_ CFU/g	9.08	(Fixed)
RES_vitro_^*a*^	Residual variability – *in vitro*	log_10_ CFU/mL	0.877	(4.9)
RES_vivo_^*a*^	Residual variability – *in vivo*	log_10_ CFU/g	0.555	(4.2)
Immune response				
*F*_33591_	Inoculum bioavailability in immunocompetent mice (*S. aureus* ATCC 33591)	–^*c*^	0.437	(6.2)
*N*_circ,T0_	Baseline circulating neutrophil count	log_10_ N/mL	6.23	(Fixed)
MTT	Neutrophil maturation mean transit time	h	37.7	(Fixed)
*N*_amp_	Surge amplitude	–	44.2	(Fixed)
*N*_T0_	Surge peak time	h	50.5	(Fixed)
*N*_SW_	Surge width	h	28.2	(Fixed)
*k*_phag,max_	Maximum phagocytosis rate constant^*b*^	h^−1^	0.963	(2.0)
*k*_dig_	Digestion rate constant^*b*^			
P/N_50_	Phagocytosed bacteria to neutrophil count ratio reducing the phagocytosis rate by 50%	CFU/N	114	(16)
*H*	Saturation sigmoidicity factor	–	0.475	(12)
Afabicin desphosphono effect				
*E*_max vitro_	Maximum afabicin desphosphono killing rate constant – *in vitro*	h^−1^	2.99	(5.2)
Ratio_Emax_	Ratio for *E*_max_ *in vivo* (*E*_max vivo_ = *E*_max vitro_ • Ratio_Emax_)	–	0.0796	(9.3)
β_vitro_	EC_50_ scaling slope – *in vitro*	–	1	(Fixed)
β_vivo_	EC_50_ scaling slope – *in vivo*	–	0.182	(16)
γ	EC_50_ scaling power (EC_50_ = β • MIC^γ^)	–	0.185	(24)
Hill	*E*_max_ model sigmoidicity factor	–	0.515	(7.4)
*k*_on_	Adaptation onset rate constant	mL/(µg•h)	0.556	(25)
*k*_off_	Adaptation offset rate constant	h^−1^	0.0139	(Fixed)
AS_50_	Fraction needed to reach 50% of reduction in E_max_	–	0.784	(10)

^*a*^ RES parameters correspond to the magnitude of residual unexplained variability on the standard deviation scale.

^*b*^ Parameters sharing the same estimate.

^*c*^ –, unitless parameters.

**TABLE 2:** Observed bacterial counts in afabicin dose-ranging studies in *S. aureus* immunocompetent mouse thigh infection models^*a*^

			Median bacterial count [change from baseline] (log_10_ cfu/g)			
Strain | MIC (mg/L)	Inoculum size (log_10_ cfu/g)	Dose (mg/kg q6h)	2 h baseline	26 h	50 h	74 h
570493 | 0.004	7.36	Control	7.87	8.63 [+0.76]	–	–
		0.32	–^*c*^	8.47 [+0.6]	8.69 [+0.82]	7.91 [+0.04]
		3.2	–	7.92 [+0.05]	7.61 [−0.26]	6.08 [−1.79]
		32	–	7.96 [+0.09]	6.60 [−1.27]	–
ATCC 29213 | 0.008	7.36	Control	7.85	8.71 [+0.86]	–	–
		0.32	–	8.90 [+1.05]	–^*b*^	–^*b*^
		3.2	–	8.11 [+0.26]	8.05 [+0.20]	7.85 [+0.00]
		32	–	7.78 [−0.07]	7.31 [−0.54]	6.48 [−1.37]
ATCC 33591 | 0.015	7.20	Control	7.57	8.33 [+0.76]	–	–
		0.32	–	8.06 [+0.49]	7.93 [+0.36]	8.34 [+0.77]
		3.2	–	7.11 [−0.46]	7.13 [−0.44]	5.41 [−2.16]
		32	–	6.93 [−0.64]	6.50 [−1.07]	5.28 [−2.29]
IHMA 1074670 | 0.06	7.14	Control	7.39	8.32 [+0.93]	–	–
		0.32	–	8.37 [+0.98]	7.99 [+0.60]	8.26 [+0.87]
		3.2	–	7.92 [+0.53]	7.08 [−0.31]	7.30 [−0.09]
		32	–	7.28 [−0.11]	6.81 [−0.58]	6.59 [−0.80]

^*a*^ Times are indicated in hours after infection. Results for all dose groups and inoculum sizes can be found in Table S2.

^*b*^ Mice sacrificed at 26 h due to reaching a humane endpoint.

^*c*^ –, time points for given doses for which no data is available. This is due to either the time point not being evaluated per protocol, or not being evaluated for an ethical reason (humane endpoint).

### Model for immune response

The final model described the effect of immune response on bacterial killing by adding a phagocytosed (P) state for bacteria, with a saturable phagocytosis rate. The surge parameters for neutrophil recruitment remained fixed to the literature values (Fig. S1) (22). The maximum phagocytosis rate constant, *k*_phag,max_, was estimated at 0.963 h^−1^, and *k*_phag_ was found to reduce as the P/N ratio increased by a sigmoidal (*H* = 0.475) function with a P/N_50_ of 114 CFU/neutrophil. No saturation of the digestion process was identified. The rate constant *k*_dig_ could share the same estimate as *k*_phag,max_ without significantly worsening the model fit (ΔOFV = 2.3). Strain-specific differences in bacterial counts in control and treatment groups for *S. aureus* ATCC 33591 were best explained by a bioavailability term F_33591_ of 0.458 for the start inoculum of that strain (ΔOFV = −120). This implementation did not improve the model fit when evaluated for other strains (ΔOFV =0.23).

**Fig 2:**
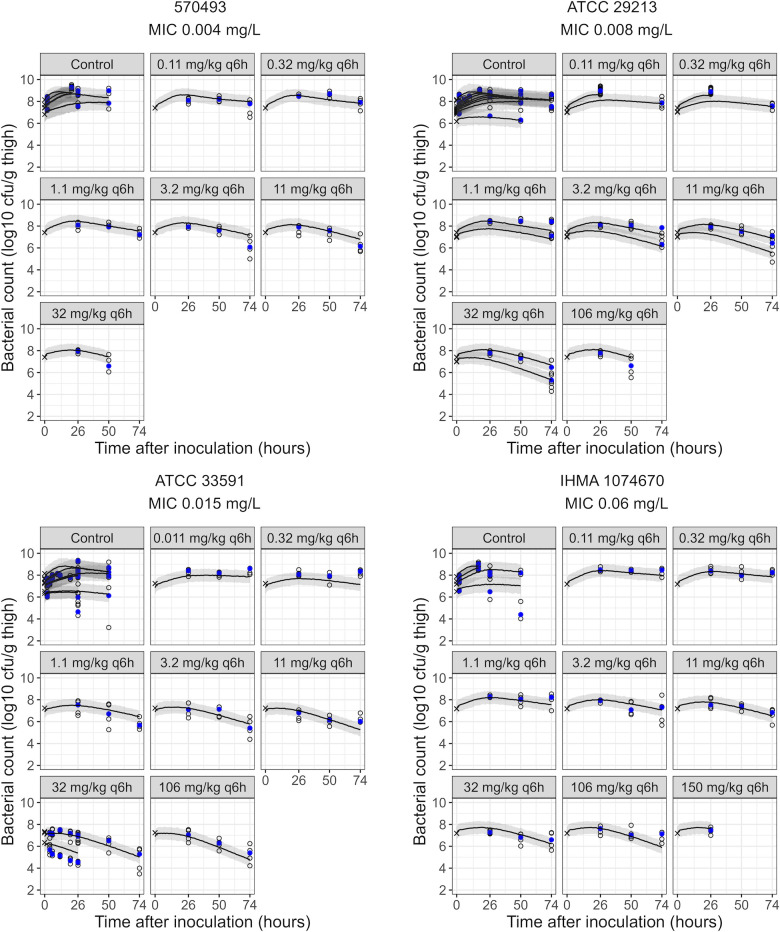
Visual predictive checks of the final model. Shown are the starting inoculum (crosses), observed (open circles), and median of observed (blue filled circles) bacterial counts, with the median (lines) and the corresponding 95% confidence interval of the median (areas) of model predictions in immunocompetent mice. Each panel presents data for one afabicin dosing regimen against one of the studied *S. aureus* strains. Separate medians of observations and prediction intervals are presented for groups with different start inocula. For *S. aureus* ATCC 33591, crosses indicate the observed inoculum, while model predictions start at the model-estimated start inoculum.

The model described growth control data across strains and inoculum sizes and fitted the treatment groups well after adding the drug effect model (Fig. 2 and Fig. S2). Pronounced variability was, however, observed in control groups with the lowest inoculum sizes (~6 log_10_ CFU/g), for which observations at 50 h after infection spanned a range of up to 4 log_10_ CFU/g. The model predicted a slow decrease of bacterial counts for the low inoculum sizes, but since parameters for variability in immune response between experiments or animals were not considered, the model could not capture all observations.

### PKPD modeling of afabicin desphosphono activity

**Fig 3:**
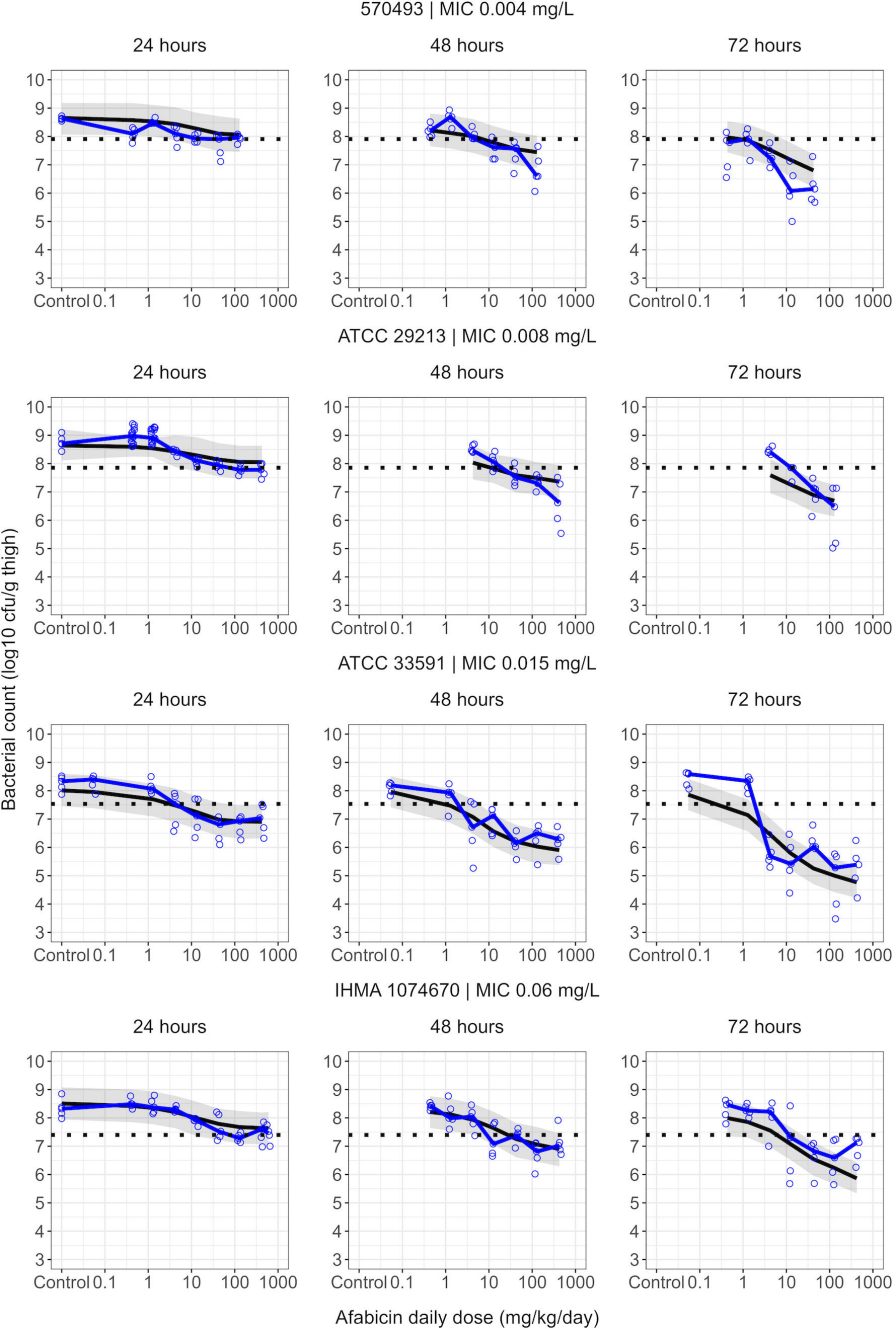
Visual predictive checks of dose-response for afabicin in immunocompetent mice at 24, 48, or 72 h after the start of treatment for the studied afabicin dose range against the evaluated *S. aureus* strains. Shown are the observed (open circles) and median of observed (blue lines) bacterial counts and the median (black lines) and 95% confidence interval of the median (areas) of model predictions based on the final model. The horizontal dashed line represents stasis for a given strain (median of bacterial counts at start of treatment from control groups with the same inoculum size).

The model structure developed based on *in vitro* and *in vivo* neutropenic mouse thigh infection data (28) could describe bacterial dynamics when used jointly with the model developed for immune response. As for neutropenic data, EC_50_ was estimated to be lower *in vivo* than *in vitro***,** with a β_vivo_ of 0.182, representing an ~80% lower EC_50_ in immunocompetent mice. Additionally, a lower maximum killing rate constant was found under immunocompetent conditions, corresponding to an *E*_max_ of 0.24 h^−1^ versus 2.99 h^−1^ *in vitro* and 3.37 h^−1^ previously estimated in neutropenic conditions (28). Other drug effect parameters were shared across the three experimental settings. All parameters were estimated with acceptable precision (relative standard errors ≤25%, Table 1). VPCs indicated that the final model could describe bacterial dynamics over 74 h of infection in most groups (Fig. 2). Nonetheless, an overestimation of bacterial counts for the lower inoculum size of the 32 mg/kg q6h groups for *S. aureus* ATCC 33591 should be noted. The model could well characterize the observed afabicin dose-response (Fig. 3). However, some mispredictions were observed, such as an underestimation of drug effect at 72 h after the start of treatment for higher doses against infections caused by *S. aureus* 570-493.

### Prediction of clinical efficacy

**Fig 4:**
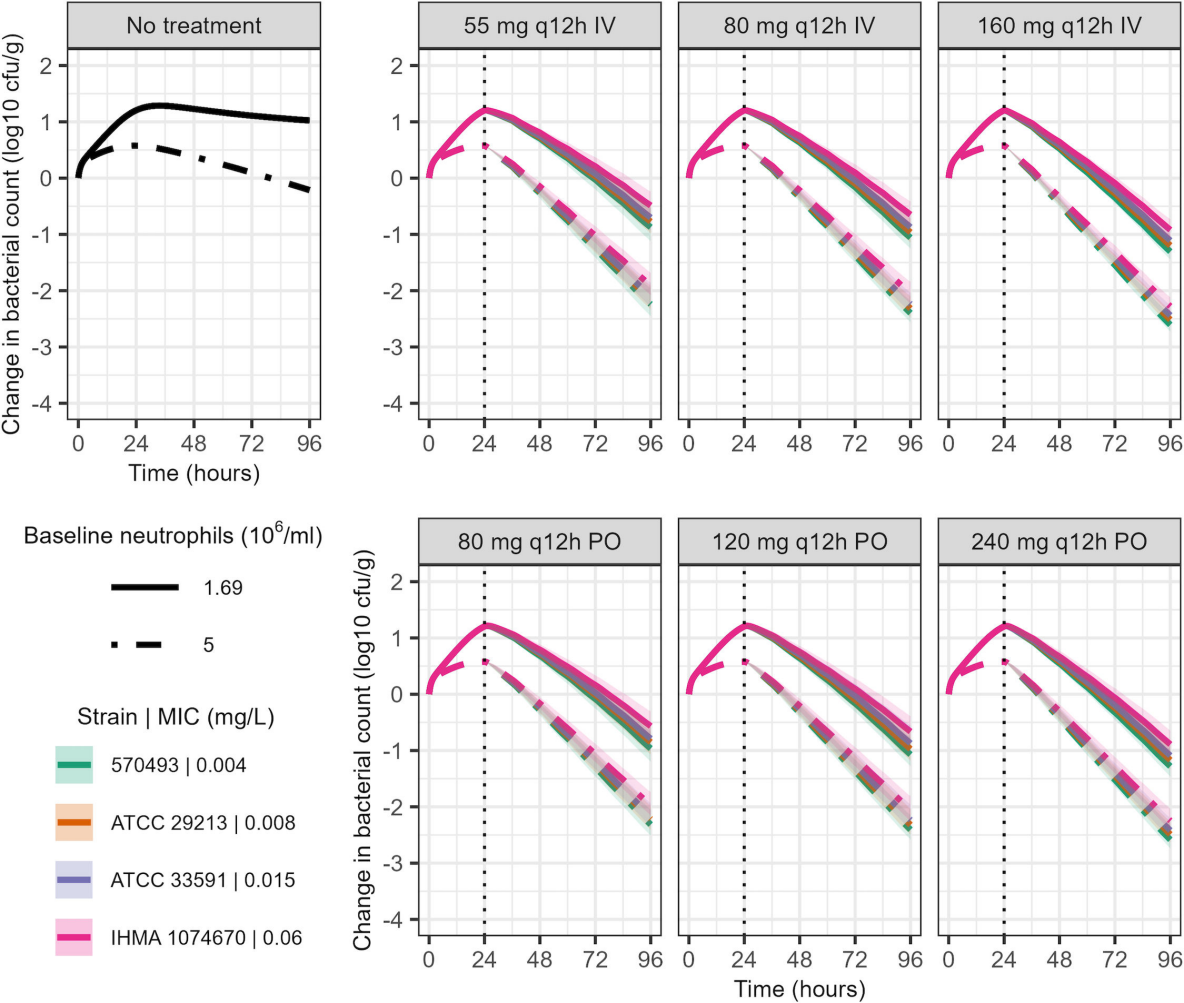
Model-predicted bacterial dynamics in immunocompetent patients, with a baseline neutrophil count of 1.69 × 10^6^/mL or 5 × 10^6^/mL and infected with an initial bacterial count of 7 log_10_ CFU/g, receiving twice-daily afabicin IV or oral administration. Shown are the median (lines) 5^th^ and 95^th^ percentiles (areas) of predicted bacterial counts from 500 simulated PK profiles per dosing regimen, *S. aureus* strain, and baseline neutrophil count. The vertical dashed line indicates treatment initiation 24 h after infection.

The predicted changes in bacterial counts for immunocompetent patients receiving twice-daily IV or oral afabicin doses are shown in Fig. 4 and Fig. S3 and S4. For both baseline neutrophil counts explored and the four evaluated strains, all simulated afabicin dosing regimens were predicted to achieve a sustained bacterial killing over 72 h of treatment, including when the variability in PK profiles was considered. A similar extent of bacterial killing was not achieved with the immune response alone.

## DISCUSSION

The present study expanded the model-based translation framework for afabicin through the development of a PKPD model characterizing both immune response and antibiotic effect in an *in vivo* immunocompetent mouse thigh infection model, utilized for predictions of efficacy in immunocompetent patients receiving various dosing regimens.

Immune response was characterized by a model describing a saturable phagocytosis process by the host’s neutrophils, consistent with previous studies (6, 11–13, 22). The estimated maximum phagocytosis rate constant of 0.963 h^−1^ was similar to the literature estimate of 0.82 h^−1^ for the same process *in vivo* in a model based on data from *Acinetobacter baumannii* lung infection in mice (22). This phagocytosis process was, however, comparatively lower in the current study when related to bacterial growth, with a growth rate constant of 1.1 h^−1^ compared to 0.502 h^−1^ in the literature model. The structure and estimates of the saturation process also differed between the present study and the literature model, which could be partially explained by multiple factors. First, neutrophil dynamics (i.e., baseline levels, maturation, and recruitment to the infection site) were fixed in the present study as neutrophil counts were unavailable. Differences in neutrophil dynamics could thus impact estimates of parameters related to saturation, which rely on infection site neutrophil counts. Additionally, infection site baseline neutrophil counts and post-infection recruitment might differ between thigh and lung infection models, and immune response parameter estimates could differ across bacterial species and strains. The present study focused on *S. aureus* infections, a pathogen known to evade the innate immune system through multiple pathways, such as interference with opsonization or the production of proteins to resist neutrophil killing (40, 41). Indeed, in a previous study, the maximal killing rate induced by granulocytes in a mouse thigh infection was 10-fold lower for a *S. aureus* strain than for a *Pseudomonas aeruginosa* strain (11). The P/N_50_ estimate of 114 may also be increased by the action of other cell types, such as macrophages, and other killing mechanisms, such as neutrophil extracellular traps, which were not described in the model. A strain-specific difference in immune response was identified for one strain, described by a bioavailability term that captures lower bacterial counts at early time points in both control and treatment groups. These trends were not observed for the other strains in immunocompetent conditions, nor for the same strain *in vitro* or in a neutropenic mouse thigh infection model (28). Through the neutrophil recruitment process and the delay between phagocytosis and digestion, the model structure allowed for capturing the observed delay in immune response. Observed differences in bacterial counts between growth control groups of different inoculum sizes were also captured. However, high variability was observed for low inoculum sizes.

The PKPD model structure previously developed for afabicin based on *in vitro* time-kill data and neutropenic mouse thigh infection data (28) was successfully combined with an immune response model to describe bacterial counts in afabicin-treated immunocompetent mice. As previously identified for neutropenic mice, EC_50_ was lower *in vivo* than *in vitro*; however, a lower *in vivo E*_max_ of 0.24 h^−1^ was estimated for afabicin desphosphono in immunocompetent mice, which was not found in neutropenic conditions with the same strains (28). This *E*_max_ value is of similar magnitude to that found in a model for tedizolid accounting for immune response (0.104 h^−1^) against *S. aureus* ATCC 33591 (6), a strain also used for PKPD model development in this analysis. A lower *E*_max_ in immunocompetent mice could be related to several factors. PKPD models for antibiotics in neutropenic conditions do not usually consider the activity of remaining immune cells. Furthermore, because establishing a stable infection was required for PKPD analyses, higher inoculum sizes were needed in immunocompetent conditions, which could lead to an inoculum effect not fully accounted for by the structure of the model, thereby reducing the observed antibiotic activity. For instance, a study of polymyxin B in a neutropenic mouse thigh infection model quantified a reduction of 32% of the drug effect slope when increasing the inoculum size from 5 to 7 log_10_ CFU/thigh (42).

The developed PKPD model could describe bacterial dynamics and capture afabicin dose-response in immunocompetent mice over 72 h of treatment. The use of an immunocompetent mouse infection model allowed for the exploration of bacterial dynamics beyond the 24-hour endpoint, which is typically used in neutropenic infection models, notably due to challenges in maintaining the neutropenic infection model for extended time durations. It should nonetheless be noted that bacterial dynamics during the first 24 h differed greatly from those observed for the same strains in a neutropenic mouse thigh infection model (28). This is likely due to the influence of the immune system, therefore limiting the comparability of results from neutropenic and immunocompetent models with simpler analysis methods. However, the model only considered an effect of afabicin desphosphono on extracellular bacteria. Afabicin desphosphono was shown to accumulate in macrophages while remaining active against intracellular *S. aureus* (8). It could be hypothesized that a fraction of phagocytosed bacteria could resist immune killing through immune evasion (40) and be affected by afabicin desphosphono. These processes could not be described in the model, as no observation of bacteria and afabicin desphosphono concentrations inside immune cells was available. Moreover, the PK model used in the analysis was developed from data in infected mice rendered neutropenic (31), while there may be PK differences in immunocompetent infected mice.

The developed model was applied to predict efficacy in patients for the different doses used in clinical studies of afabicin. Under all simulation conditions, the tested IV or oral afabicin doses were predicted to induce a sustained reduction of bacterial counts for all studied *S. aureus* strains over 3 days of treatment, assuming neutrophils would be as potent in humans as in mice. Depending on the baseline neutrophil counts, a 1- or 2-log bacterial kill was achieved over the treatment duration. A reduction of bacterial counts at 72 h after the start of treatment, matching the timing of lesion response evaluation, may be associated with an observed reduction of the lesion size in the clinical setting. Additionally, minimal differences in outcomes were predicted between the evaluated dosing regimens. Overall, these predictions are in agreement with the results of the Phase II study in ABSSSI (27), in which both low (IV 80 mg twice-daily followed by oral 120 mg twice-daily) and high (IV 160 mg twice-daily followed by oral 240 mg twice-daily) dose afabicin regimens were non-inferior to vancomycin/linezolid, with comparable early clinical response rates for both dosing regimens (94.6% and 90.1% for low and high dose groups, respectively) in the microbiological intent to treat population. These results highlight how the model predictive performance may benefit from accounting for the immune response for predictions in non-neutropenic populations. The model also predicted efficacy for the lower dosing regimens of 55 mg IV q12h and 80 mg PO q12h, which were not tested in the ABSSSI Phase 2 trial. These dosing regimens are currently evaluated in a Phase 2 trial in BJI and have shown encouraging preliminary results (43). Of note, limitations of the current simulations are potential PK differences between patients and healthy volunteers, from whom the PK model was built. A different drug clearance would result in different exposure and potential differences in killing. The predictions also did not consider differences in immune response parameters between mice and humans. Additionally, although mouse thigh infection models are commonly used and recommended in the translation of antibiotic PKPD (1, 44), there may be differences associated with the infection site (thigh infections against ABSSSI or BJI), which were not considered in simulations. These assumptions imply that the simulations may not accurately predict bacterial dynamics in patients if there are significant discrepancies, e.g., in bacterial killing by the immune system between the studied animal model and patients. Nonetheless, these limitations should be considered in light of current standards. The PK/PD indices, commonly used for antibiotic PKPD translation, rely on several assumptions and limitations, such as their single time point nature, the use of summary PK metrics and of the MIC as the sole PD marker, and their inability to account for the dynamics of the immune response (15).

In conclusion, a PKPD model successfully describing bacterial counts over time in an immunocompetent mouse thigh infection model was developed. The model was able to characterize bacterial killing attributable to both immune response and afabicin and was subsequently used to predict the clinical efficacy of different afabicin dosing regimens in immunocompetent patients. This study illustrates how a model-based translation framework, spanning from the initial use of *in vitro* time-kill data to clinical predictions in immunocompetent conditions, could be applied in antibiotic drug development to inform study design and decision-making at each phase, ultimately improving the efficiency of antibiotic drug development.

## SUPPLEMENTAL MATERIAL

Supplemental material aac.00959-25-s0001.docx Tables S1 to S3; Fig. S1 to S4.

ASM does not own the copyrights to Supplemental Material that may be linked to, or accessed through, an article. The authors have granted ASM a non-exclusive, world-wide license to publish the Supplemental Material files. Please contact the corresponding author directly for reuse.
