# Supplementary material for: Model-based quantification of immune response and anti-staphylococcal activity of afabicin in immunocompetent mouse thigh infections to enable predictions of clinical efficacy
Source: Antimicrob Agents Chemother. 2026 Mar 4;70(4):e00959-25. doi: 10.1128/aac.00959-25 (PMC13041350; doi:10.1128/aac.00959-25)
Supplement: Supplemental material — Tables S1 to S3; Fig. S1 to S4. [file aac.00959-25-s0001.docx]

**Supplementary text 1.** Neutrophil dynamics from the final model

Neutrophil dynamics were described by the following equations:

$$\frac{\text{dN}_{\text{prol}}}{\text{dt}}\text{=-}\text{k}_{\text{tr}}\text{∙}\text{N}_{\text{prol}}\text{+}\text{k}_{\text{tr}}\text{∙}\text{N}_{\text{prol}}\text{∙}\left( \frac{\text{N}_{\text{circ,T0}}}{\text{N}_{\text{circ}}} \right)^{\text{0.149}}$$

$$\frac{\text{dN}_{\text{tr1}}}{\text{dt}}\text{=}\text{ -}\text{k}_{\text{tr}}\text{∙}\text{N}_{\text{tr1}}\text{+}\text{k}_{\text{tr}}\text{∙}\text{N}_{\text{prol}}$$

$$\frac{\text{dN}_{\text{tr2}}}{\text{dt}}\text{=}\text{-}\text{k}_{\text{tr}}\text{∙}\text{N}_{\text{tr2}}\text{+}\text{k}_{\text{tr}}\text{∙}\text{N}_{\text{tr1}}$$

$$\frac{\text{dN}_{\text{tr3}}}{\text{dt}}\text{=}\text{-}\text{k}_{\text{tr}}\text{∙}\text{N}_{\text{tr3}}\text{+}\text{k}_{\text{tr}}\text{∙}\text{N}_{\text{tr2}}$$

$$\frac{\text{dN}_{\text{circ}}}{\text{dt}}\text{=}\text{-}\text{k}_{\text{tr}}\text{∙}\text{N}_{\text{circ}}\text{+}\text{k}_{\text{tr}}\text{∙}\text{N}_{\text{tr3}}$$

$$\frac{\text{dN}_{\text{thigh}}}{\text{dt}}\text{=-}\text{k}_{\text{tr}}\text{∙}\text{N}_{\text{thigh}}\text{+}\text{k}_{\text{tr}}\text{∙}\left[ \text{1+}\frac{\text{N}_{\text{amp}}}{\left( \frac{\text{T-}\text{N}_{\text{T0}}}{\text{N}_{\text{sw}}} \right)^{\text{6}}\text{+1}} \right]\text{∙}\text{N}_{\text{circ}}$$

With k_tr_ the transit rate constant defined as 4/MTT (mean transit time), N_prol_ the proliferative cells, N_circ,T0_ the baseline circulating neutrophil count, N_circ_ the circulating neutrophil count, N_tr1-3_ the transit compartments for neutrophil maturation, N_thigh_ the thigh neutrophil count, N_amp_ the surge amplitude parameter, N_T0_ the surge peak time, N_SW_ the surge width parameter and T the system time. All compartments were initialised to N_circ,T0_ at time = 0 h.

**Supplementary text 2.** PK model for afabicin in mice

Afabicin desphosphono concentrations following afabicin intraperitoneal administration were derived using the following equations:

$$\frac{\text{dDepot}}{\text{dt}}\text{=}\text{ -}\text{k}_{\text{t}}\text{∙Depot}$$

$$\frac{\text{dTransit}}{\text{dt}}\text{=}\text{ -}\text{k}_{\text{t}}\text{∙Transit+}\text{k}_{\text{t}}\text{∙Depot}$$

$$\frac{\text{dCentral}}{\text{dt}}\text{=}\text{ -}\text{k}_{\text{e}}\text{∙Central-}\text{k}_{\text{cp}}\text{∙Central+}\text{k}_{\text{pc}}\text{∙}\text{Periph}\text{+}\text{k}_{\text{a}}\text{∙}\text{Transit∙Conv}$$

$$\frac{\text{dPeriph}}{\text{dt}}\text{=}\text{ }\text{k}_{\text{cp}}\text{∙Central-}\text{k}_{\text{pc}}\text{∙}\text{Periph}$$

With Depot the depot compartment, Transit the absorption transit compartment, Central the central compartment, Periph the peripheral compartment, k_t_ the transit rate constant, k_a_ the absorption rate constant, Conv the conversion factor from afabicin to the active metabolite afabicin desphosphono, k_e_ the elimination rate constant, k_cp_ the distribution from Central to Periph rate constant, and k_pc_ the distribution from Periph to Central rate constant. Unbound concentrations from Central were used to drive the drug effect in the PKPD model.

**Table S1.** List of *S. aureus* strains studied in the immunocompetent mouse thigh infection model.

| Strain name | Relevant Resistance phenotype | Afabicin desphosphono MIC (mg/L) | Growth control groups | Afabicin-treated groups |
| --- | --- | --- | --- | --- |
| 570-493 | MSSA | 0.004 | X | X |
| ATCC 29213 | MSSA | 0.008 | X | X |
| ATCC 33591 | MRSA | 0.015 | X | X |
| IHMA 1073118 | MRSA | 0.03 | X |  |
| IHMA 1074670 | MRSA | 0.06 | X | X |

**Table S2.** Observed bacterial counts in afabicin dose-ranging studies in *S. aureus* immunocompetent mouse thigh infection models. Times are indicated in hours after infection.

|  |  |  | Median bacterial count [change from baseline] (log_10_ cfu/g) | | | | | |
| --- | --- | --- | --- | --- | --- | --- | --- | --- |
| Strain \| MIC (mg/L) | Inoculum size  (log_10_ cfu/g) | Dose  (mg/kg q6h) | 2h baseline | 26h | | 50h | | 74h |
| 570493 \| 0.004 | 7.36 | Control | 7.87 | 8.63  [+0.76] | | - | | - |
|  |  | 0.11 | - | 8.10 [+0.23] | | 8.19 [+0.32] | | 7.78 [-0.09] |
|  |  | 0.32 | - | 8.47 [+0.6] | | 8.69 [+0.82] | | 7.91 [+0.04] |
|  |  | 1.1 | - | 8.10 [+0.23] | | 7.94 [+0.07] | | 7.23 [-0.64] |
|  |  | 3.2 | - | 7.92 [+0.05] | | 7.61 [-0.26] | | 6.08 [-1.79] |
|  |  | 11 | - | 7.90 [+0.03] | | 7.57 [-0.30] | | 6.15 [-1.72] |
|  |  | 32 | - | 7.96 [+0.09] | | 6.60 [-1.27] | | - |
|  | 6.77 | Control | 7.23 | 7.58 [+0.35] | | 7.83 [+0.60] | | - |
|  | 7.4 | Control | 7.94 | 8.98 [+1.04] | | 8.98 [+1.04] | | - |
|  |  | | 2h baseline | 21h^a^ | |  | |  |
|  | 7.67 | Control | 8.30 | 9.27 [+0.87] | | - | | - |
|  | 8.11 | Control | 8.39 | 9.27 [+0.88] | | - | | - |
| Strain \| MIC (mg/L) | Inoculum size  (log_10_ cfu/g) | Dose  (mg/kg q6h) | 2h baseline | 26h | | 50h | | 74h |
| ATCC 29213 \| 0.008 | 7.36 | Control | 7.85 | 8.71 [+0.86] | | - | | - |
|  |  | 0.11 | - | 8.98 [+1.13] | | - | | - |
|  |  | 0.32 | - | 8.90 [+1.05] | | -^a^ | | -^a^ |
|  |  | 1.1 | - | 8.42 [+0.57] | | 8.46 [+0.61] | | 8.41 [+0.56] |
|  |  | 3.2 | - | 8.11 [+0.26] | | 8.05 [+0.20] | | 7.85 [+0.00] |
|  |  | 11 | - | 7.93 [+0.08] | | 7.51 [-0.34] | | 7.10 [-0.75] |
|  |  | 32 | - | 7.78 [-0.07] | | 7.31 [-0.54] | | 6.48 [-1.37] |
|  |  | 106 | - | 7.78 [-0.07] | | 6.62 [-1.23] | | - |
|  | 6.97 | Control | 7.85 | - | | - | | 7.51 [-0.34] |
|  |  | 0.11 | - | - | | - | | 7.87 [+0.02] |
|  |  | 0.32 | - | - | | - | | 7.60 [-0.25] |
|  |  | 1.1 | - | - | | - | | 7.1 [-0.75] |
|  |  | 3.2 | - | - | | - | | 6.32 [-1.53] |
|  |  | 11 | - | - | | - | | 6.44 [-1.41] |
|  |  | 32 | - | - | | - | | 5.31 [-2.54] |
|  | 6.13 | Control | 6.89 | 6.67 [-0.22] | | 6.30 [-0.59] | | - |
|  | 6.80 | Control | 7.57 | 8.41 [+0.84] | | 7.87 [+0.30] | | - |
|  | 7.05 | Control | 7.51 | 7.88 [+0.37] | | 7.55 [+0.04] | | - |
|  | 7.17 | Control | 7.71 | 8.82 [+1.11] | | 8.65 [+0.94] | | - |
|  | 8.10 | Control | 8.59 | - | | 8.95 [+0.36] | | 8.61 [+0.02] |
|  |  | | 2h baseline | 6h | | 10h | |  |
|  | 7.23 | Control | 7.72 | 8.19 [+0.47] | | 8.47 [+0.75] | | - |
|  |  | | 2h baseline | 18h^a^ | | -^a^ | |  |
|  | 7.54 | Control | 7.93 | 9.04 [+1.11] | | - | | - |
| Strain \| MIC (mg/L) | Inoculum size  (log_10_ cfu/g) | Dose  (mg/kg q6h) | 2h baseline | 26h | | 50h | | 74h |
| ATCC 33591 \| 0.015 | 7.20 | Control | 7.57 | 8.33 [+0.76] | | - | | - |
|  |  | 0.011 | - | 8.40 [+0.83] | | 8.19 [+0.62] | | 8.59 [+1.02] |
|  |  | 0.32 | - | 8.06 [+0.49] | | 7.93 [+0.36] | | 8.34 [+0.77] |
|  |  | 1.1 | - | 7.53 [-0.04] | | 6.72 [-0.85] | | 5.68 [-1.89] |
|  |  | 3.2 | - | 7.11 [-0.46] | | 7.13 [-0.44] | | 5.41 [-2.16] |
|  |  | 11 | - | 6.81 [-0.76] | | 6.15 [-1.42] | | 6.02 [-1.55] |
|  |  | 32 | - | 6.93 [-0.64] | | 6.50 [-1.07] | | 5.28 [-2.29] |
|  |  | 106 | - | 7.04 [-0.53] | | 6.28 [-1.29] | | 5.39 [-2.18] |
|  |  | | 2h baseline | 4h | 6h | 12h | 20h | 26h |
|  | 6.27 | Control | 6.06 | - | - | - | - | 5.98 [-0.08] |
|  |  | 32 | - | 5.69 [-0.07] | 5.28 [-0.78] | 5.13 [-0.93] | 4.70 [-1.36] | 4.54 [-1.52] |
|  | 7.28 | Control | 7.15 | 7.29 [+0.14] | 7.63 [+0.49] | 8.0 [+0.85] | 7.77 [+0.62] | 7.86 [+0.71] |
|  |  | 32 | - | 7.19 [+0.04] | 7.16 [+0.01] | 7.49 [+0.34] | 7.19 [+0.04] | 7.0 [-0.15] |
|  |  | | 2h baseline | 26h | | 50h | | 74h |
|  | 6.48 | Control | 6.36 | 4.65 [-1.71] | | 6.13 [-0.23] | | - |
|  | 7.26 | Control | 7.09 | 8.04 [+0.95] | | 7.89 [+0.80] | | - |
|  | 7.67 | Control | 7.31 | 8.41 [+1.10] | | 8.28 [+0.97] | | - |
|  | 8.11 | Control | 7.59 | 9.29 [+1.70] | | 8.70 [+1.11] | | - |
|  |  | | 2h baseline | 6h | | 10h | |  |
|  | 7.58 | Control | 7.67 | 7.98 [+0.31] | | 8.13 [+0.46] | | - |
| IHMA 1074670 \| 0.06 | 7.14 | Control | 7.39 | 8.32 [+0.93] | | - | | - |
|  |  | 0.11 | - | 8.48 [+1.09] | | 8.43 [+1.04] | | 8.46 [+1.07] |
|  |  | 0.32 | - | 8.37 [+0.98] | | 7.99 [+0.60] | | 8.26 [+0.87] |
|  |  | 1.1 | - | 8.29 [+0.90] | | 8.04 [+0.65] | | 8.22 [+0.83] |
|  |  | 3.2 | - | 7.92 [+0.53] | | 7.08 [-0.31] | | 7.30 [-0.09] |
|  |  | 11 | - | 7.53 [+0.14] | | 7.38 [-0.01] | | 6.84 [-0.55] |
|  |  | 32 | - | 7.28 [-0.11] | | 6.81 [-0.58] | | 6.59 [-0.80] |
|  |  | 106 | - | 7.60 [+0.21] | | 7.02 [-0.37] | | 7.13 [-0.26] |
|  |  | 150 | - | 7.46 [+0.07] | | - | | - |
|  | 6.46 | Control | 6.58 | 6.48 [-0.10] | | 4.40 [-2.18] | | - |
|  | 7.13 | Control | 7.42 | 8.01 [+0.59] | | 8.22 [+0.80] | | - |
|  |  | | 2h baseline | 17h^a^ | | -^a^ | |  |
|  | 7.4 | Control | 7.68 | 8.57 [+0.89] | | - | | - |
|  | 7.83 | Control | 8.00 | 9.01 [+1.01] | | - | | - |
| Strain \| MIC (mg/L) | Inoculum size  (log_10_ cfu/g) | Dose  (mg/kg q6h) | 2h baseline | 26h | | 50h | | 74h |
| IHMA 1073118 \| 0.03 | 6.2 | Control | 6.51 | 5.80 [-0.71] | | 5.27 [-1.24] | | - |
|  | 6.72 | Control | 7.42 | 8.02 [+0.60] | | 8.23 [+0.81] | | - |
|  | 7.1 | Control | 7.39 | 8.40 [+1.01] | | 8.02 [+0.63] | | - |
|  |  | | 2h baseline | 18h^a^ | | -^a^ | |  |
|  | 7.56 | Control | 7.88 | 8.87 [+0.99] | | - | | - |

^a^Mice sacrificed at an earlier timepoint due to reaching a humane endpoint.

**Table S3.** Parameters of the afabicin human PK model used for simulation.

| Parameter | Description | Units | Value | IIV variance |
| --- | --- | --- | --- | --- |
| CL_P_ | Afabicin clearance | L/h | 31.4 | 0.0579 |
| V_p_ | Afabicin central volume of distribution | L | 4.42 | - |
| CLD1_P_ | Afabicin intercompartmental clearance 1 | L/h | 1.02 | - |
| VP1_P_ | Afabicin peripheral volume 1 | L | 0.723 | 0.0419 |
| CLD2_P_ | Afabicin intercompartmental clearance 2 | L/h | 0.335 | 0.424 |
| VP2_P_ | Afabicin peripheral volume 2 | L | 33 | - |
| CL_BL_ | Afabicin desphosphono baseline clearance | L/h | 3.85 | 0.104 |
| HTCL_BL_ | Height effect on afabicin desphosphono baseline clearance | L/h/cm | 0.0459 | - |
| CL_max_ | Maximal increase in afabicin desphosphono clearance | L/h | 1.35 | 0.358 |
| K_out_ | Rate of time-increase in clearance | h^-1^ | 0.0368 | 0.831 |
| V | Afabicin desphosphono central volume | L | 31.4 | 0.275 |
| WTV | Weight effect on afabicin desphosphono central volume | L/kg | 0.573 | - |
| CLD | Afabicin desphosphono intercompartmental clearance | L/h | 30.1 | 1.05 |
| VP | Afabicin desphosphono peripheral volume | L | 27.1 | - |
| F_oral_ | Bioavailability (logit) for oral administration | - | 1.69 | 0.335 |
| DF_oral_ | Power for dose effect on bioavailability logit | - | 0.246 | - |
| K_a_ | Absorption rate constant (oral administration) | h^-1^ | 1.01 | 1.35 |
| K_t_ | Absorption transit rate constant (Oral administration, fasted conditions) | h^-1^ | 2.61 | 0.779 |
| MWR | Molecular weight ratio | - | 0.773 | - |


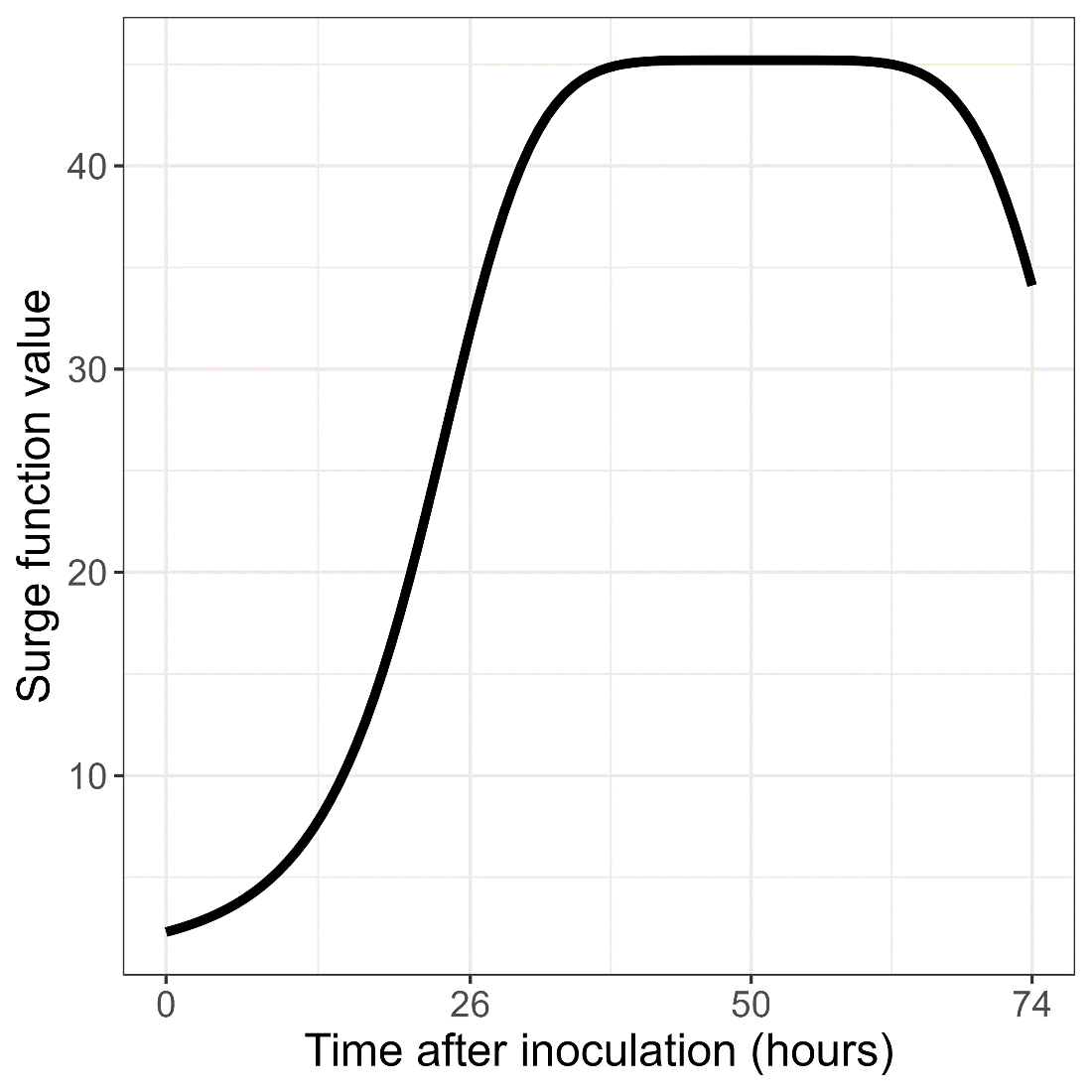


**Figure S1.** Graphical representation of the surge function for neutrophil recruitment to the infection site over time. The surge begins at time of infection.


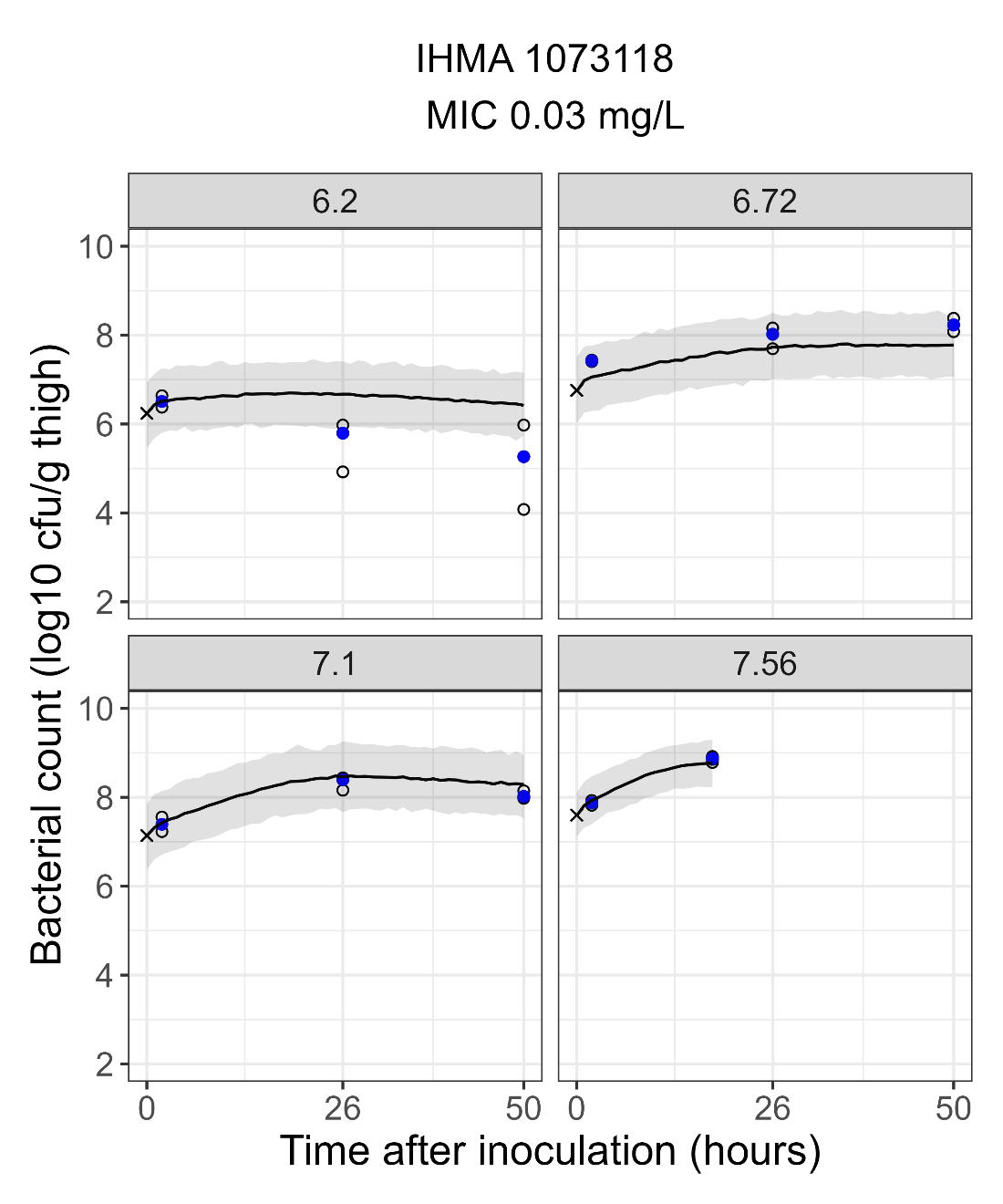


**Figure S2.** Visual predictive check (VPC) of the final model for growth control data of *S. aureus* IHMA 1073118, stratified by start inoculum size (in log10 cfu/g). Shown are the starting inoculum (crosses), observed (open circles) and median of observed (blue filled circles) bacterial counts, with the median (lines) and the corresponding 95% confidence interval of the median (areas) of model predictions in immunocompetent mice.


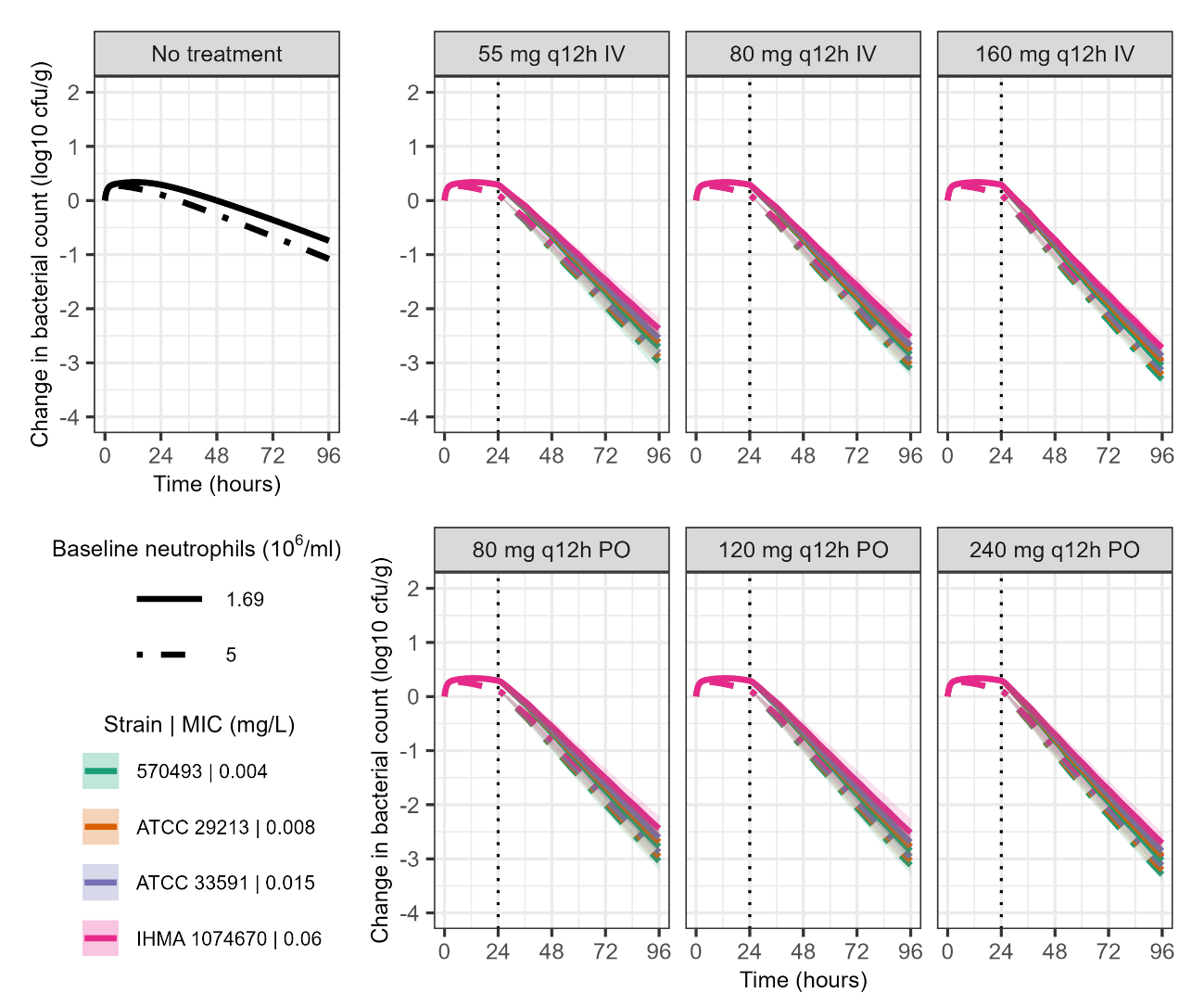


**Figure S3.** Model-predicted bacterial dynamics in immunocompetent patients, with a baseline neutrophil count of 1.69x10^6^ /ml or 5x10^6^ /ml and infected with an initial bacterial count of 6 log_10_ CFU/g, receiving twice-daily afabicin IV or oral administration. Shown are the median (lines), 5^th^ and 95^th^ percentiles (areas) of predicted bacterial counts from 500 simulated PK profiles per dosing regimen, *S. aureus* strain and baseline neutrophil count. The vertical dashed line indicates treatment initiation 24 hours after infection.

**
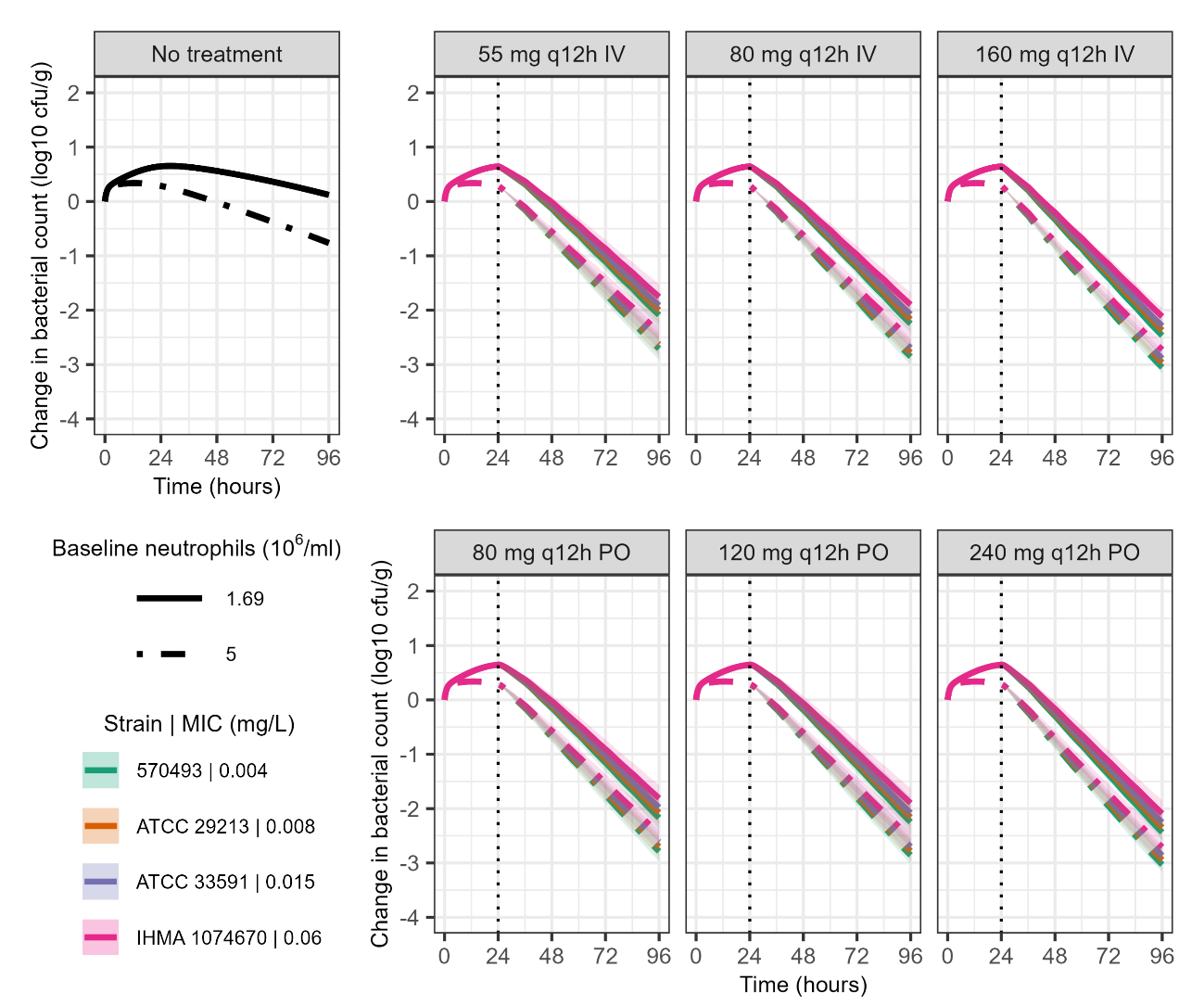
Figure S4.** Model-predicted bacterial dynamics in immunocompetent patients, with a baseline neutrophil count of 1.69x10^6^ /ml or 5x10^6^ /ml and infected with an initial bacterial count of 6.5 log_10_ CFU/g, receiving twice-daily afabicin IV or oral administration. Shown are the median (lines), 5^th^ and 95^th^ percentiles (areas) of predicted bacterial counts from 500 simulated PK profiles per dosing regimen, *S. aureus* strain and baseline neutrophil count. The vertical dashed line indicates treatment initiation 24 hours after infection.
